# Bioinformatics analysis of the microRNA genes associated with type 2 cardiorenal syndrome

**DOI:** 10.1186/s12872-024-03816-z

**Published:** 2024-03-05

**Authors:** Yani Zong, Yuexin Hu, Mengdi Zheng, Zhi Wang

**Affiliations:** 1 Department of Cardiovascular Medicine, Affiliated Nanjing Brain Hospital, Nanjing Medical University, Nanjing, China; 2 Department of Cardiovascular Medicine, Nanjing Chest Hospital, Nanjing, China; 3 Department of Cardiology Affiliated Nanjing Brain Hospital, Nanjing Medical University, No. 264 Guangzhou Road, Nanjing, Jiangsu, 210029 China

**Keywords:** Cardiorenal syndrome, MicroRNAs, Bioinformatics

## Abstract

**Background:**

MicroRNAs (miRNAs) are important regulatory factors in the normal developmental stages of the heart and kidney. However, it is currently unclear how miRNA is expressed in type 2 cardiorenal syndrome (CRS). This study aimed to detect the differential expression of miRNAs and to clarify the main enrichment pathways of differentially expressed miRNA target genes in type 2 CRS.

**Methods:**

Five cases of healthy control (Group 1), eight of chronic heart failure (CHF, Group 2) and seven of type 2 CRS (Group 3) were enrolled, respectively. Total RNA was extracted from the peripheral blood of each group. To predict the miRNA target genes and biological signalling pathways closely related to type 2 CRS, the Agilent miRNA microarray platform was used for miRNA profiling and bioinformatics analysis of the isolated total RNA samples.

**Results:**

After the microarray analysis was done to screen for differentially expressed circulating miRNAs among the three different groups of samples, the target genes and bioinformatic pathways of the differential miRNAs were predicted. A total of 38 differential miRNAs (15 up- and 23 down-regulated) were found in Group 3 compared with Group 1, and a total of 42 differential miRNAs (11 up- and 31 down-regulated) were found in Group 3 compared to Group 2. According to the Gene Ontology (GO) function and Kyoto Encyclopaedia of Genes and Genomes (KEGG) pathway analysis, the top 10 lists of molecular functions, cellular composition and biological processes, and the top 30 signalling pathways of predicted gene targets of the differentially expressed miRNAs were discriminated among the three groups.

**Conclusion:**

Between the patients with CHF and type 2 CRS, miRNAs were differentially expressed. Prediction of target genes of differentially expressed miRNAs and the use of GO function and KEGG pathway analysis may reveal the molecular mechanisms of CRS. Circulating miRNAs may contribute to the diagnosis of CRS, and further and larger studies are needed to enhance the robustness of our findings.

## Introduction

Cardiorenal syndrome (CRS) is a pathological condition in which acute or chronic insufficiency of the heart or kidney leads to equally acute or chronic dysfunction of the other organ. Given the complexity of the pathogenesis and clinical presentation of CRS, Ronco et al. have further divided CRS into five subtypes [1]. Of these, type 2 CRS is defined as progressive renal failure caused by chronic abnormalities of cardiac function. The highest incidence of type 2 CRS, ranging from 45 to 63%, accompanied by an increase in mortality and disability rates [2, 3].

Recently, the study of epigenetic biomarkers has resulted in the discovery of numerous drugs that play an increasingly significant role in clinical therapy, especially in treating cancer and cardiovascular diseases [4]. This advancement has propelled traditional medicine into the realm of precision medicine. The research on non-coding RNAs, particularly microRNAs (miRNAs), is a hot topic in the field of epigenetic markers. MicroRNAs are a class of single-stranded, small, endogenous non-coding RNAs consisting of 18−24 nucleotides that regulate gene expression at the post-transcriptional level by targeting the 3′-untranslated region of bound messenger RNAs (mRNAs) and completely or incompletely complementarily binding target gene mRNAs, thereby inhibiting the translation of protein-coding mRNAs [5, 6]. There is extensive research evidence that miRNA expression is involved in the development and progression of cardiovascular and renal disease. For example, microRNA-21 (miR-21) exhibits dysregulation in various heart and kidney diseases. It has repeatedly been suggested as a therapeutic target for treating CRS [7]. However, the role of miRNAs in the occurrence and development of CRS is unclear [8–10]. This study applied bioinformatics analysis to detect the differential expression of miRNAs and to clarify the main enrichment pathways of target genes of differentially expressed miRNAs in type 2 CRS.

## Materials and methods

### Study subjects

**Fig. 1:**
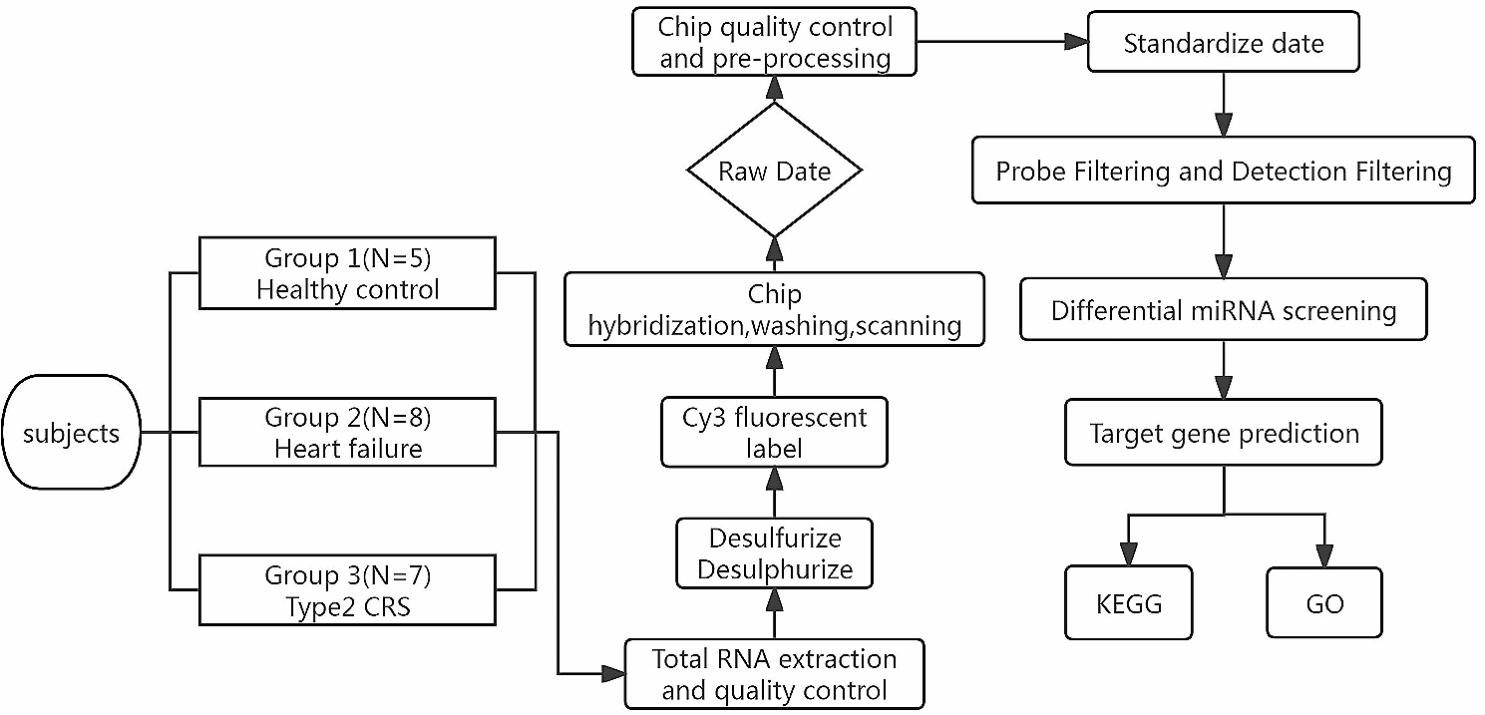
Flowchart of the current study. (CRS, cardiorenal syndrome; GO, Gene Ontology; KEGG, Kyoto Encyclopaedia of Genes and Genomes)

The flowchart of the current study is illustrated in Fig. 1. In this study, 20 subjects were enrolled, including 5 healthy controls (Group 1), 8 patients with chronic heart failure (CHF) and normal renal function (Group 2) and 7 patients with type 2 CRS (Group 3). The diagnosis of CHF was based on the ESC guidelines for the diagnosis and treatment of acute and chronic heart failure [11]. All patients with CHF had clinical signs and symptoms of CHF, and all had a reduced left ventricular ejection fraction (LVEF≤ 40%). The estimated glomerular filtration rate (eGFR) was calculated according to the modified Modification of Diet in Renal Disease (MDRD) formula for the Chinese population (eGFR = 175 × [creatinine (mg/dL)]^−1.234^ × [age (years)]^−0.179^ × sex (male = 1, female = 0.79) [12]. Type 2 CRS was diagnosed according to a Scientific Statement of Cardiorenal Syndrome from the American Heart Association [13]. Exclusion criteria were as follows: primary renal disease, type 1 or type 2 diabetes, pregnancy, primary liver disease, autoimmune diseases, haematological disorders, tumours and underlying diseases that significantly affect cardiac and renal function, such as hyperthyroidism, hypothyroidism, primary aldosteronism and other endocrine disorders. All recruited subjects signed an informed consent form. The study was approved by the Ethics Committee of Nanjing Brain Hospital. The study protocol was in accordance with the Declaration of Helsinki.

### Blood biochemical tests

Fasting venous blood was taken from each subject early in the morning of the day following enrolment. Routine blood tests were performed on a fully automated haematology analyser (Roche, USA). Serum lipid profile and liver and kidney function were measured with an Olympus automatic analyser (Olympus, Japan). Serum NT-proBNP levels were measured by electrochemical methods using an Elecsys 2010 analyser (Roche).

### Echocardiography

All subjects underwent cardiac examination by colour Doppler imaging (Hewlett-Packard, CA, USA). The left ventricular end-systolic diameter (LVDs), left ventricular end-diastolic diameter (LVDd), left atrial diameter (LAD) and right atrial diameter (RAD) were measured on the long axis two-chamber section of the left ventricle. The LVEF was measured by the Simpson method. Each parameter was tested 3 times, and the average value was determined.

### Extraction of total RNA

Plasma samples of each subject were collected and lysed using erythrocyte lysate. Total RNA was isolated using TRIzol (Invitrogen), quantified using a NanoDrop ND-2000 (Thermo Scientific) and assayed by the Agilent Bioanalyzer 2100 (Agilent Technologies).

### MiRNA detection and data analysis

After RNA quality control, samples were labelled and hybridised to the Agilent Human miRNA Microarray Kit, Release 21.0 (8 × 60 K; DesignID:070156) containing 2570 probes for mature miRNAs and eluted according to the standard microarray procedure. After elution, the original images were scanned using the Agilent Scanner G2505C (Agilent Technologies). Data analysis of the samples was performed at Shanghai Ouyi Biomedical Technology (Shanghai, China). The raw images were processed using Feature Extraction software (version 10.7.1.1; Agilent Technologies) to extract the raw data. Before conducting further analysis, data pre-processing is imperative. This involves the essential task of cleaning and standardising the data to eliminate any noise and outliers. Common pre-processing methods include data cleaning, missing value processing, and data standardisation. Data cleaning involves removing outliers and erroneous data by filtering and refining the dataset. Missing value processing involves utilizing interpolation, deletion, or replacement methods to manage missing data points effectively. Conversely, standardization of data encompasses converting data into a standardised format with a zero mean and one variance. This facilitates subsequent statistical analysis and model building. At least one set of probes marked as “detected” in each group of samples used for comparison was left for subsequent analysis. Differential miRNAs were screened using *p*-values and fold change (FC) values from *t*-tests, with the screening criteria being up- or down-regulated FC values ≥ 2.0 and *p*-values ≤ 0.05, while scatter plots and volcano plots (corresponding to groupings with biological replicates) were plotted for each set of differential screening results.

### Gene Ontology (GO) function and Kyoto Encyclopaedia of genes and genomes (KEGG) pathway analysis

Target gene prediction was performed for the differential miRNAs using the miRWalk and miRDB databases, and GO and KEGG enrichment analyses were performed on the predicted target genes to determine the biological functions or pathways that the differentially expressed miRNAs might affect. The GO database contains three main categories: Biological Process (BP) represents a process of molecular activity events, including a functional collection of cells, tissues, organs and species; Cellular Component (CC) represents a cell or its external environment; Molecular Function (MF) is a description of the active components of gene products at the molecular level. KEGG is a database for systematic analysis of gene function, linking genomic and functional information. This enrichment analysis was based on all pathways in the KEGG pathway database. Pathway analysis of differentially expressed genes was performed using the KEGG data, and the significance of enrichment of differential genes in each pathway was calculated using the hypergeometric distribution algorithm.

### Statistical analysis

GraphPad Prism 8 software was used for data analysis. Continuous normally distributed variables were expressed as mean ± standard deviation. Inter-group comparisons were analysed using an unpaired *t*-test. The measurements of non-normally distributed data were expressed using the median (Q1, Q3). The counting data were expressed as a percentage and analysed using a chi-square test. A *p*-value < 0.05 was considered statistically significant.

## Results

### Characteristics of clinical baseline information

As shown in Table 1, there was no significant difference in age or gender among the three groups. All patients with CHF and those with type 2 CRS had dilated cardiomyopathy as their underlying heart disease. Compared with the control and CHF groups, the serum creatinine level in the type 2 CRS group was significantly increased, while Cys-C and eGFR were significantly reduced. Compared with the control group, both the CHF and type 2 CRS groups showed a significant increase in NT-proBNP and a significant decrease in LVEF.

**Table 1:** The baseline characteristics of patients and controls

Factors	Group 1 (Control, *N* = 5)	Group 2 (CHF, *N* = 8)	Group 3 (Type 2 CRS, *N* = 7)
Male (%)	2(40%)	6 (75%)	6(85.7%)
Female (%)	3(60%)	2 (25%)	1(14.3%)
Age	70.6 ± 4.5	62.9 ± 3.3	56.7 ± 6.9
SBP(mmHg)	138.4 ± 3.0	129.9 ± 5.0	150.4 ± 19.0
DBP(mmHg)	73.2 ± 5.9	80.4 ± 2.9	91.3 ± 13.7
Etiology			
DCM (%)	0	8 (100%)	7 (100%)
Laboratory			
BUN (mmol/L)	5.26 ± 0.8	6.65 ± 0.3	9.64 ± 2.7
Cr (umol/L)	61.4 ± 5.2	80.8 ± 4.2*	133.7 ± 13.9*#
Cys-c(mg/L)	0.96 ± 0.1	0.99 ± 0.1	1.50 ± 0.2*#
eGFR (ml/min/1.73m^2^)	141.6 ± 13.4	110.0 ± 5.9*	67.2 ± 5.6*#
NT-proBNP (pg/mL)	58.83 (48.51, 122.7)	1915 (573.6, 3221)*	2452 (1375, 9407)*
Echocardiogram			
LAD (mm)	42.2 ± 2.1	47.9 ± 2.2	47.4 ± 2.3
RAD (mm)	36.2 ± 2.7	41.5 ± 2.7	47.4 ± 3.3*
LVDs (mm)	31.6 ± 1.0	54.8 ± 2.0*	51.7 ± 3.9*
LVDd (mm)	47.6 ± 1.7	67.9 ± 2.2*	70.4 ± 2.5*
LVEF (%)	62.0 ± 0.4	38.0 ± 1.8*	41.0 ± 2.8*

vs. Group 1, * *p* < 0.05; vs. Group 2, # *p* < 0.05

Abbreviations: CHF, chronic heart failure; CRS, cardiorenal; SBP, systolic blood pressure; DBP, diastolic blood pressure; DCM, dilated cardiomyopathy; BUN, blood urea nitrogen; Cr, creatinine; Cys-c, Cystatin C; eGFR, estimated glomerular filtration rate; NT-proBNP, N-terminal pro-brain natriuretic peptide; LAD, left atrial diameter; RAD, right atrial diameter; LVDs, left ventricular end systolic diameter; LVDd, left ventricular end diastolic diameter; LVEF, left ventricular ejection fraction

### Differential miRNA screening among the three groups

**Fig. 2:**
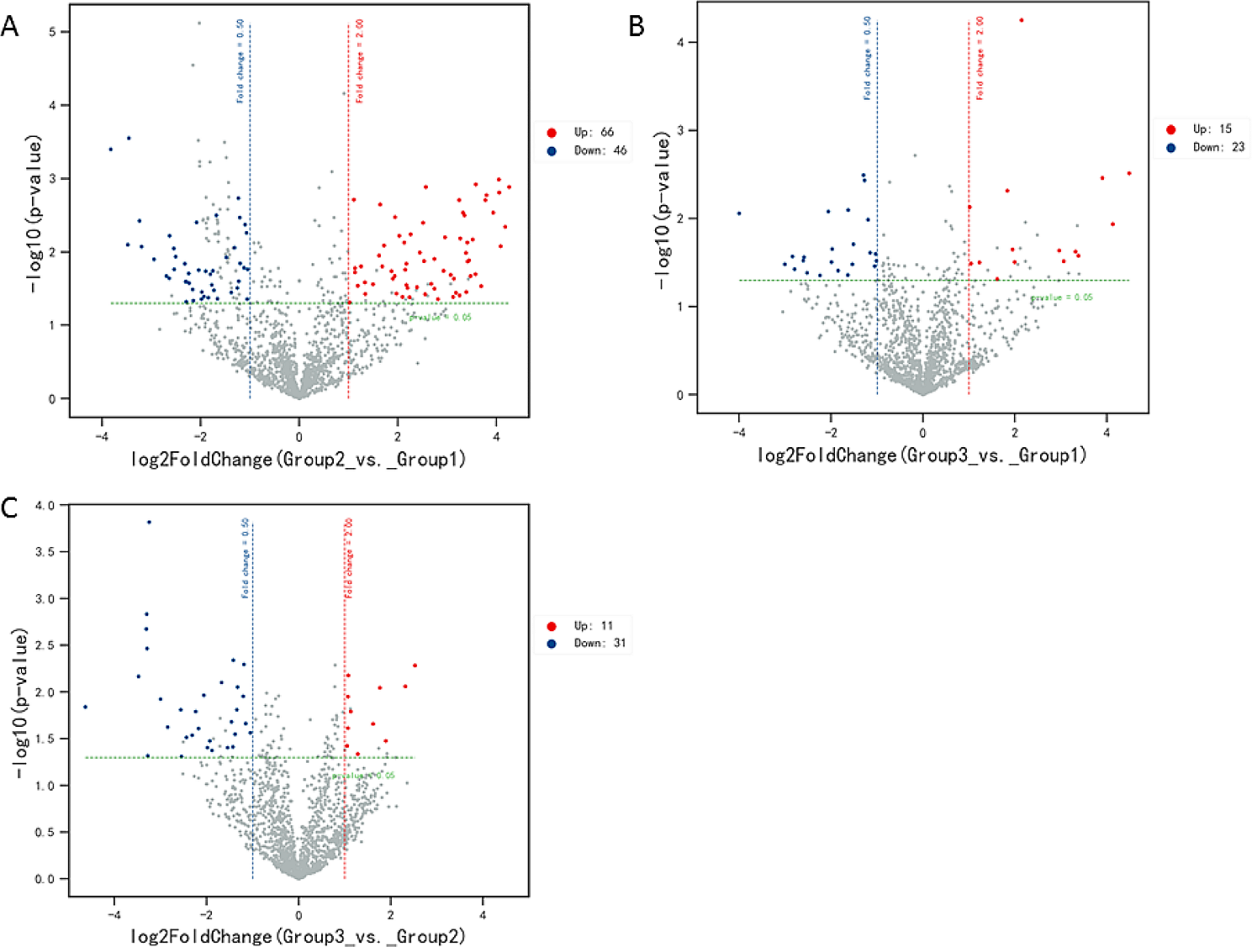
Volcano map of differentially expressed miRNAs among the three groups. (up, up-regulated; down, down-regulated; Red and blue dots indicate up-regulated and down-regulated miRNAs, respectively.)

As shown in Fig. 2, there were 112 differentially expressed miRNAs (66 up- and 46 down-regulated) in Group 2 compared to Group 1, 38 differentially expressed miRNAs (15 up- and 23 down-regulated) in Group 3 compared to Group 1, and 42 differentially expressed miRNAs (11 up- and 31 down-regulated) in Group 3 compared to Group 2. The detailed information about the miRNAs is shown in Table 2.

**Table 2:** Differential microRNA screening among the three groups

Name	MicroRNAs	
Group2 VS Group1	UP	hsa-miR-129-5p,hsa-miR-432-5p,hsa-miR-518c-5p,hsa-miR-516b-5p,hsa-miR-10a-3p,hsa-miR-127-5p,hsa-miR-491-3p,hsa-miR-551b-5p,hsa-miR-1208,hsa-miR-1295a,hsa-miR-1261,hsa-miR-1321,hsa-miR-3121-3p,hsa-miR-3124-5p,hsa-miR-4324,hsa-miR-4260,hsa-miR-4325,hsa-miR-4269,hsa-miR-4289,hsa-miR-3617-5p,hsa-miR-3654,hsa-miR-3660,hsa-miR-3666,hsa-miR-3678-3p,hsa-miR-4436a,hsa-miR-4448,hsa-miR-4450,hsa-miR-4451,hsa-miR-4458,hsa-miR-4531,hsa-miR-3189-5p,hsa-miR-4648,hsa-miR-4654,hsa-miR-4660,hsa-miR-4676-5p,hsa-miR-4694-3p,hsa-miR-4736,hsa-miR-4749-5p,hsa-miR-4753-5p,hsa-miR-4436b-3p,hsa-miR-5003-5p,hsa-miR-5187-5p,hsa-miR-5195-5p,hsa-miR-5572,hsa-miR-5584-5p,hsa-miR-1295b-3p,hsa-miR-5708,hsa-miR-513c-3p,hsa-miR-1237-5p,hsa-miR-6074,hsa-miR-6500-3p,hsa-miR-6720-3p,hsa-miR-598-5p,hsa-miR-1266-3p,hsa-miR-513b-3p,hsa-miR-6726-5p,hsa-miR-6754-5p,hsa-miR-6801-3p,hsa-miR-6839-5p,hsa-miR-7151-3p,hsa-miR-7155-3p,hsa-miR-7157-5p,hsa-miR-7157-3p,hsa-miR-7854-3p,hsa-miR-8055,hsa-miR-8088
	DOWN	hsa-miR-425-3p,hsa-miR-191-3p,hsa-miR-563,hsa-miR-634,hsa-miR-643,hsa-miR-454-5p,hsa-let-7b-3p,hsa-let-7f-1-3p,hsa-miR-221-5p,hsa-miR-1229-3p,hsa-miR-1237-3p,hsa-miR-1238-3p,hsa-miR-1825,hsa-miR-1539,hsa-miR-3180-5p,hsa-miR-4310,hsa-miR-4313,hsa-miR-4274,hsa-miR-3679-3p,hsa-miR-3162-3p,hsa-miR-4664-3p,hsa-miR-4728-3p,hsa-miR-4731-3p,hsa-miR-4749-3p,hsa-miR-4787-3p,hsa-miR-5690,hsa-miR-1304-3p,hsa-miR-98-3p,hsa-miR-4750-3p,hsa-miR-6508-5p,hsa-miR-1908-3p,hsa-miR-6731-3p,hsa-miR-6752-3p,hsa-miR-6757-3p,hsa-miR-6775-3p,hsa-miR-6795-3p,hsa-miR-6796-3p,hsa-miR-6798-3p,hsa-miR-6812-3p,hsa-miR-6813-3p,hsa-miR-6824-3p,hsa-miR-6861-3p,hsa-miR-6880-3p,hsa-miR-6890-3p,hsa-miR-7114-3p,hsa-miR-8485
Group3 VS Group2	UP	hsa-miR-34a-5p,hsa-miR-550a-3p,hsa-miR-595,hsa-miR-625-5p,hsa-miR-627-5p,hsa-miR-106a-3p,hsa-let-7i-3p,hsa-miR-1825,hsa-miR-500b-5p,hsa-miR-5690,hsa-miR-6865-3p
	DOWN	hsa-miR-100-5p,hsa-miR-520b,hsa-miR-518c-5p,hsa-miR-516b-5p,hsa-miR-10a-3p,hsa-miR-127-5p,hsa-miR-508-5p,hsa-miR-518e-5p,hsa-miR-1323,hsa-miR-1208,hsa-miR-378c,hsa-miR-3617-5p,hsa-miR-3659,hsa-miR-4444,hsa-miR-4450,hsa-miR-4458,hsa-miR-4488,hsa-miR-4514,hsa-miR-4648,hsa-miR-4725-3p,hsa-miR-4748,hsa-miR-5003-3p,hsa-miR-1295b-3p,hsa-miR-513c-3p,hsa-miR-6720-3p,hsa-miR-1266-3p,hsa-miR-513b-3p,hsa-miR-6754-5p,hsa-miR-6873-5p,hsa-miR-7157-3p,hsa-miR-8088
Group3 VS Group1	UP	hsa-miR-129-5p,hsa-miR-625-5p,hsa-miR-92a-1-5p,hsa-miR-491-3p,hsa-miR-1261,hsa-miR-4269, hsa-miR-4289,hsa-miR-3660,hsa-miR-4448,hsa-miR-4451,hsa-miR-4736,hsa-miR-4753-5p,hsa-miR-5696,hsa-miR-6753-3p,hsa-miR-8077
	DOWN	hsa-miR-133b,hsa-miR-520b,hsa-miR-663a,hsa-miR-18b-3p,hsa-miR-1237-3p,hsa-miR-3180-5p,hsa-miR-4274,hsa-miR-3610,hsa-miR-3620-3p,hsa-miR-3648,hsa-miR-3679-3p,hsa-miR-3937,hsa-miR-4417,hsa-miR-4508,hsa-miR-4716-5p,hsa-miR-4740-5p,hsa-miR-4787-3p,hsa-miR-4750-3p,hsa-miR-1908-3p,hsa-miR-6796-3p,hsa-miR-6798-3p,hsa-miR-6824-3p,hsa-miR-7114-3p

### Differential miRNA target gene prediction among the three groups

In this study, the miRWalk and miRDB databases were used to predict up- and down-regulated miRNAs as possible target genes to be regulated, and the intersecting genes appearing in these 2 databases were finally obtained for subsequent analysis. The database intersection analysis predicted a total of 4069 differential miRNA target genes (2555 up- and 1514 down-regulated) when comparing Group 2 to Group 1, a total of 905 differential miRNA target genes (488 up- and 417 down-regulated) in the comparison between Group 3 and Group 1, and a total of 1506 differential miRNA target genes (263 up-regulated and 1243 down-regulated) for Group 3 compared to Group 2.

### GO enrichment analysis of differential genes among the three groups

**Fig. 3:**
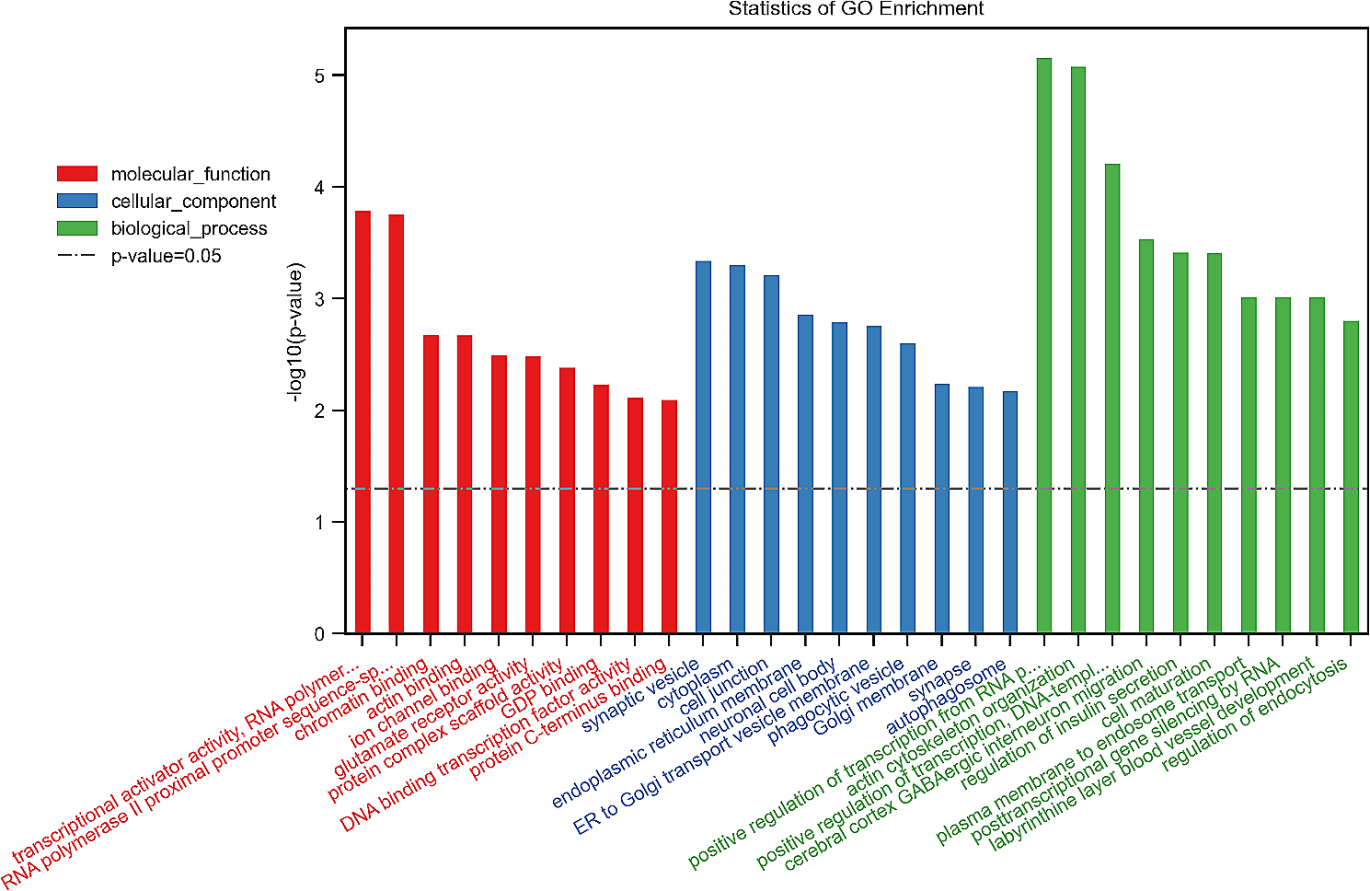
GO enrichment analysis of differential genes between Group 3 and 1. (The Y-axis in the graph is the negative logarithm of *p* value, and the higher the height of the bar graph, the smaller the corresponding *p* value. Different colour distributions correspond to BP, CC, and MF.)

**Fig. 4:**
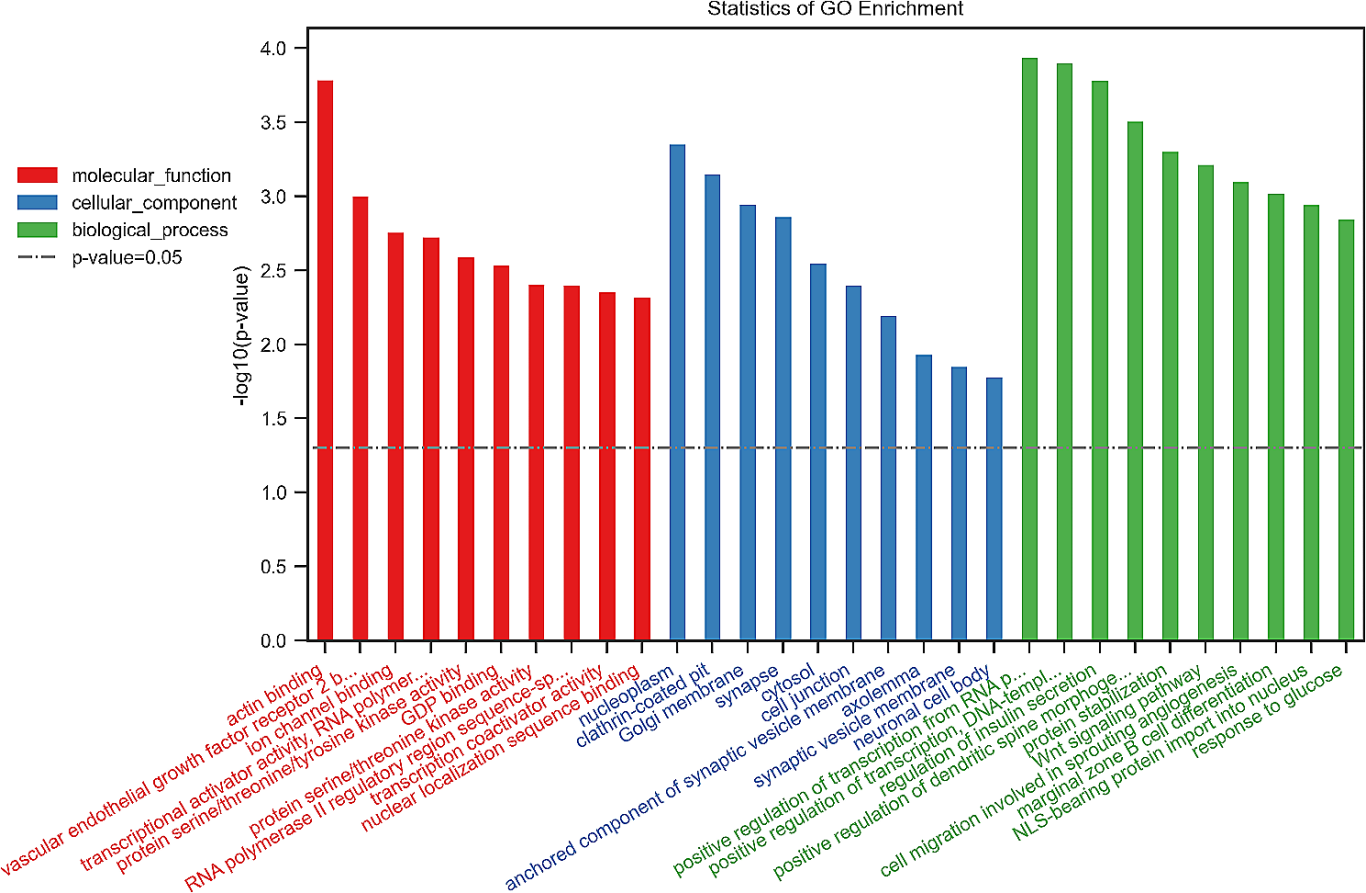
GO enrichment analysis of differential genes between Group 3 and 2. (The Y-axis in the graph is the negative logarithm of *p*-value, and the higher the height of the bar graph, the smaller the corresponding *p*-value. Different colour distributions correspond to BP, CC, and MF.)

The top 10 BPs, CCs and MFs corresponding to the differential target genes between Group 3 and Group 1 are shown in Fig. 3. The top 10 BPs included (1) positive regulation of transcription from RNA polymerase II promoter, (2) actin cytoskeleton organisation, (3) positive regulation of transcription DNA-templated, (4) cerebral cortex GABAergic interneuron migration, (5) regulation of insulin secretion, (6) cell maturation, (7) plasma membrane to endosome transport, (8) post-transcriptional gene silencing by RNA, (9) labyrinthine layer blood vessel development, (10) regulation of endocytosis; the top 10 CCs included (1) synaptic vesicle, (2) cytoplasm, (3) cell junction, (4) endoplasmic reticulum membrane, (5) neuronal cell body, (6) ER to Golgi transport vesicle membrane, (7) phagocytic vesicle, (8) Golgi membrane, (9) synapse, (10) autophagosome; and the top 10 MFs included (1) transcriptional activator activity, (2) RNA polymerase II proximal promoter sequence-specific DNA binding, (3) chromatin binding, (4) actin binding, (5) ion channel binding, (6) glutamate receptor activity, (7) molecular function protein complex scaffold activity, (8) GDP binding, (9) DNA-binding transcription factor activity, (10) protein C-terminus binding. In the differential gene enrichment analysis between Group 3 and Group 2 (Fig. 4), the top 10 BPs included (1) positive regulation of transcription from RNA polymerase II promoter, (2) positive regulation of transcription DNA-templated, (3) regulation of insulin secretion, (4) positive regulation of dendritic spine morphogenesis, (5) protein stabilisation, (6) Wnt signalling pathway, (7) cell migration involved in sprouting angiogenesis, (8) marginal zone B cell differentiation, (9) NLS-bearing protein import into nucleus, (10) response to glucose; the top 10 CCs included (1) nucleoplasm, (2) clathrin-coated pit, (3) Golgi membrane, (4) synapse, (5) cytosol, (6) cell junction, (7) anchored component of synaptic vesicle membrane, (8) axolemma, (9) synaptic vesicle membrane, (10) neuronal cell body; and the top 10 MFs included (1) actin binding, (2) vascular endothelial growth factor receptor 2 binding, (3) ion channel binding, (4) transcriptional activator activity, RNA polymerase II proximal promoter sequence-specific DNA binding, (5) protein serine/threonine/tyrosine kinase activity, (6) GDP binding, (7) protein serine/threonine kinase activity, (8) RNA polymerase II regulatory region sequence-specific DNA binding, (9) transcription coactivator activity, (10) nuclear localisation sequence binding.

### KEGG enrichment analysis of differential genes among the three groups

**Fig. 5:**
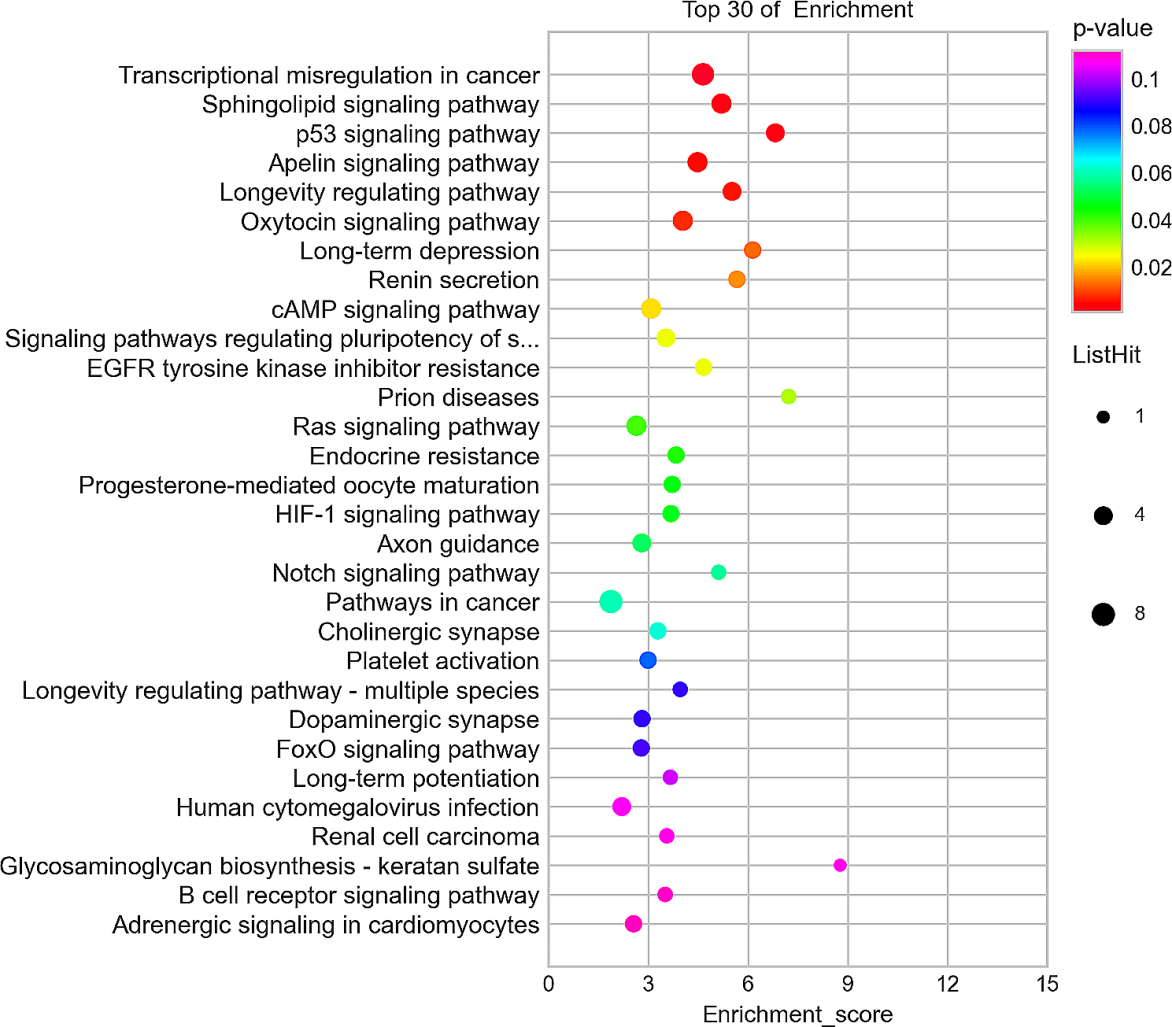
Top 30 most significantly up-regulated enriched KEGG signalling pathways between Group 3 and 2. (KEGG bubble chart is depicted, where the X-axis represents the enrichment degree, and the Y-axis denotes the enriched pathways. Larger dots on the graph indicate a higher number of genes in each pathway, with the colour of the bubbles transitioning from purple to blue, green, and finally red. A smaller enrichment *p*-value indicates greater significance.)

**Fig. 6:**
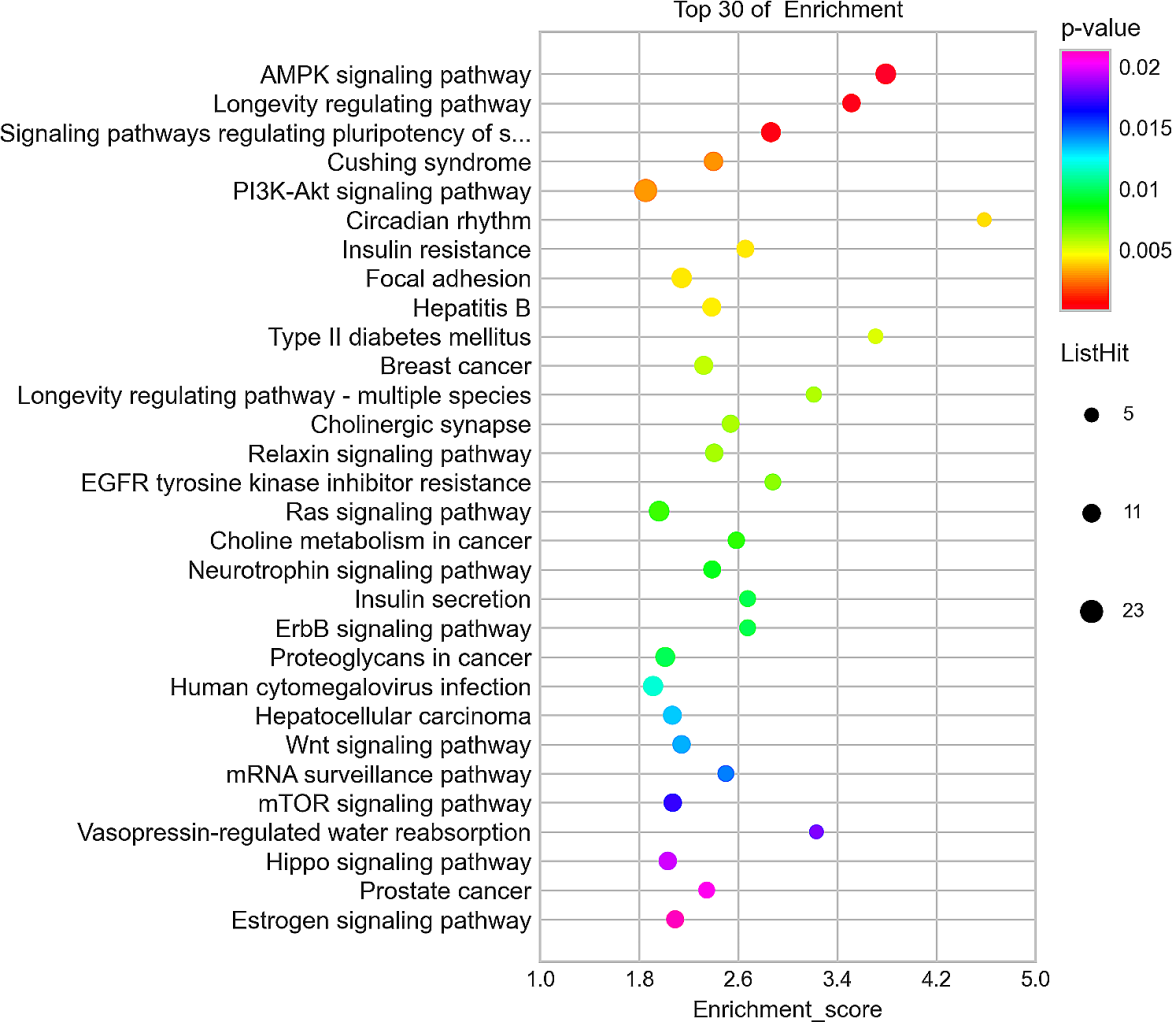
Top 30 most significantly down-regulated enriched KEGG signalling pathways between Group 3 and 2. (KEGG bubble chart is depicted, where the X-axis represents the enrichment degree, and the Y-axis denotes the enriched pathways. Larger dots on the graph indicate a higher number of genes in each pathway, with the colour of the bubbles transitioning from purple to blue, green, and finally red. A smaller enrichment *p*-value indicates greater significance.)

The differential enrichment analysis between Group 3 and Group 1 showed that the top 30 most significantly enriched KEGG signalling pathways included (1) proteoglycans in cancer, (2) long-term potentiation, (3) parathyroid hormone synthesis, secretion and action, (4) morphine addiction, (5) adenylate-activated protein kinase (AMPK) signalling pathway, (6) retrograde endocannabinoid signalling, (7) arrhythmogenic right ventricular cardiomyopathy, (8) dilated cardiomyopathy, (9) MAPK signalling pathway, (10) dopaminergic synapse, 11) oxytocin signalling pathway, 12) glutamatergic synapse, 13) Rap1 signalling pathway, 14) hypertrophic cardiomyopathy, 15) adrenergic signalling in cardiomyocytes, 16) hepatocellular carcinoma, 17) amphetamine addiction, 18) GABAergic synapse, 19) melanoma, 20) nicotine addiction, 21) focal adhesion, 22) circadian entrainment, 23) aldosterone synthesis and secretion, 24) calcium signalling pathway, 25) long-term depression, 26) ErbB signalling pathway, 27) insulin resistance, 28) Hippo signalling pathway, 29) mineral absorption, 30) SNARE interactions in vesicular transport. The differential enrichment analysis between Group 3 and Group 2 showed that the top 30 most significantly enriched KEGG signalling pathways included (1) AMPK signalling pathway, (2) longevity regulating pathway, (3) signalling pathways regulating pluripotency of stem cells, (4) EGFR tyrosine kinase inhibitor resistance, (5) longevity regulating pathway-multiple species, (6) breast cancer, (7) sphingolipid signalling pathway, (8) focal adhesion, (9) Ras signalling pathway, (10) PI3K-Akt signalling pathway, 11) transcriptional mis-regulation in cancer, 12) Cushing syndrome, 13) cholinergic synapse, 14) human cytomegalovirus infection, 15) hepatitis B, 16) hypoxia-inducible factor-1 (HIF-1) signalling pathway, 17) relaxin signalling pathway, 18) hepatocellular carcinoma, 19) proteoglycans in cancer, 20) mRNA surveillance pathway, 21) Hippo signalling pathway, 22) endocrine resistance, 23) axon guidance, 24) prostate cancer, 25) Wnt signalling pathway, 26) choline metabolism pathway, 27) choline metabolism in cancer, 28) circadian rhythm, 29) cGMP-PKG signalling pathway, 30) ErbB signalling pathway, 31) insulin secretion. In the differential enrichment analysis between Group 3 and Group 2, the top 30 most significantly up- and down-regulated enriched KEGG signalling pathways are shown in Figs. 5 and 6, respectively.

## Discussion

The search for effective prevention and treatment strategies for CRS is currently a hot topic of research. In this study, a bioinformatics approach was used to identify the differential expression of endogenous miRNAs between healthy individuals, patients with CHF and patients with type 2 CRS. The result suggests that miRNAs are widely involved in the development and progression of CRS.

Previous studies have shown that miRNA miR-34a is widely involved in reactive oxygen species (ROS) accumulation, mitochondrial dysfunction and oxidative stress [14, 15]. In the present study, the differential gene expression results showed that miR-34a expression was up-regulated in patients with CRS compared to patients with CHF. In an earlier study, it was found that down-regulation of miR-34a in rats by injection with miR-34a inhibitor before the modelling of renal ischemia-reperfusion injury could improve the renal ischemia-reperfusion-induced inflammatory response and apoptosis by increasing the level of KLF4, which led to the improvement of renal function [16]. Previously, miRNA miR-129-5p mimetic treatment was able to slow down the progression of myocardial hypertrophy and restore systolic and diastolic dysfunction in angiotensin II (AngII)-induced heart failure mice [17]. Recently, studies have shown that miR-129-5p was poorly expressed in hepatic fibrosis tissues and over-expression of miR-129-5p attenuated hepatic fibrosis through the NF-κB signalling pathway in a rat model [18].

In this study, we found that miR-129-5p was up-regulated in patients with CHF and patients with CRS. It suggests that miR-129-5p may serve as a potential therapeutic target for CHF or CRS in the future. We also found that the expression of miR-625-5p in patients with CRS was not only higher than that of healthy controls but also higher than that of patients with CHF.

Cai et al. found that miR-625-5p could attenuate AngII-induced myocardial hypertrophy through the CaMKII/STAT3 signalling pathway [19]. Abu-Halima et al. found that the expression level of miR-625-5p was lower in patients with tetralogy of Fallot who had symptomatic right heart failure, suggesting that down-regulation of miR-625-5p levels may indicate disease progression [20]. The above evidence suggests that up-regulation of miR-625-5p level plays a protective role in the organism, while down-regulation of miR-625-5p may indicate disease progression.

A large body of evidence has shown that the pathogenesis of CRS is mainly due to the adverse effects of the neurohormonal system, haemodynamics and inflammatory factors [21, 22]. The long-term hypotensive and hypoperfusion state accompanying CHF activates the renin−angiotensin−aldosterone system (RAAS) and the sympathetic nervous system (SNS) to restore tissue perfusion. After the activation of the RAAS, a large amount of AngII is synthetically released under the action of renin, which acts on the corresponding receptors in the heart and kidneys, causing cardiomyocyte hypertrophy and hyper-constriction of the renal vasculature, then renal blood flow reduces, and glomerular filtration rate decreases, triggering a number of physiological processes, such as hypoxia, inflammatory responses and, ultimately, irreversible damage to the kidneys [23].

However, recent studies have shown that oxidative stress plays an integral role in the development of CRS [21]. Oxidative stress is defined as a state of imbalance between oxidants and antioxidants, where excessive accumulation of oxidants in the body leads to cellular damage. ROS is an oxidant produced by cellular metabolism, mainly in the mitochondria, and AngII, synthesised by the over-activation of RSSA and SNS, activates NADPH-oxidase to produce ROS, which causes mitochondrial dysfunction and thus promotes oxidative damage, ultimately leading to cellular damage, endothelial cellular dysfunction and renal tubulointerstitial fibrotic alterations [24]. These lines of evidence predict a correlation between excessive accumulation of ROS and potential damage to the heart and kidney, which may have a beneficial impact on the poor prognosis of patients with CRS as a direction for new therapies.

In this study, we predicted the potential biological processes in different signalling pathways by GO and KEGG analyses among three groups. Interestingly, we found many traditional signalling pathways and receptors involved in cardiac and renal pathophysiological processes. These include signalling pathways, such as AMPK, Forkhead box-containing protein, O subfamily (FoxO), mechanistic target of rapamycin (mTOR) and HIF-1 signalling pathways, which suggests that these pathways may be related to the underlying pathophysiological mechanisms of CRS, such as activation of RAAS and SNS, inflammatory factor release and oxidative stress.

Previous studies have shown that the AMPK signalling pathway is mainly associated with oxidative stress. AMPK is a key molecule in the regulation of bioenergy metabolism and is expressed in various metabolism-related organs. It maintains energy homeostasis mainly by detecting the changes in the ATP level, and the AMPK signalling pathway is activated when the ATP level is elevated [25, 26]. Most of the ATP in the body is produced by cardiomyocytes, but in CHF, the ATP produced by cardiomyocytes will be reduced by 30−40%, which leads to the inability of the AMPK signalling pathway to be activated properly, resulting in the disruption of mitochondrial homeostasis and the generation of ROS, which ultimately causes damage to cardiac and renal cells [27–30].

Through KEGG enrichment analysis of differential genes, we found that the AMPK signalling pathway was down-regulated in patients with CRS compared to patients with CHF, which suggests that down-regulation of the AMPK signalling pathway expression may be an important underlying biological process in the development of CRS when in the CHF state.

FoxO transcription factors are a subfamily of transcriptional regulators in the Forkhead family. They have a conserved 110-amino-acid DNA-binding motif known as the “forkhead box” or “winged helix” structural domain [31]. Oxidative stress may activate FoxO1 to regulate apoptosis and autophagy-related genes, resulting in mitochondrial dysfunction, which leads to cardiomyocyte damage and, ultimately, impaired myocardial function [32]. It has also been reported that FoxO1 is involved in mediating mitochondrial oxidative stress along with putative kinase 1 (PINK1) [33]. In our observation, FoxO expression levels were up-regulated in patients with CRS compared to patients with CHF, predicting that FoxO over-expression may be involved in the pathophysiological process of CRS. In addition, we found that the HIF-1 signalling pathway was also up-regulated.

Previous studies have shown that enhancing the expression of HIF-1α can promote the metabolic transition from fatty acid oxidation to glycolysis. Intermediary metabolites of the glycolytic metabolic pathway are essential for the completion of many biological functions [34]. The mTOR is a major regulator of cellular processes, such as protein synthesis, cell growth, proliferation, autophagy, lysosomal function and cell metabolism, and activation of the mechanistic target of rapamycin complex 1 (mTORC1) enhances the expression of HIF-1 [35]. Among the above signalling pathways, the AMPK pathway has been shown to be a major upstream regulator of mTORC1. AMPK can directly inhibit the response of mTORC1 to stress, and mTORC1 activity is reduced, leading to the shutdown of anabolic processes. It was shown that in rat cardiac fibroblasts, AngII-induced cardiac fibroblast to myofibroblast transformation was associated with mTOR activation and AMPK down-regulation [36]. Moreover, mTORC1 was found to activate the transcription factor yin-yang 1 (YY1)/peroxisome proliferator-activated receptor γ coactivator 1α (PGC-1α) transcriptional complex and enhance the expression of genes involved in mitochondrial biogenesis [37]. The above evidence suggests that inhibition of mTOR expression could be a potential target for cardio-protection.

In this study, the down-regulation of mTOR level was only observed in the top 30 of the enrichment analysis comparing patients with CRS and patients with CHF, from which we can preliminarily speculate that down-regulation of the mTOR level happens during the process of disease transformation progression from CHF to CRS. Under the stimulation conditions of prolonged oxidative stress and a large amount of AngII synthesis, the expression level of mTOR is down-regulated as a self-protection strategy to delay further cardiac and renal damage. However, exploring how miRNA mediates CRS and how these signalling pathways interact with each other through in vitro and in vivo experiments warrants further studies.

The limitations of our study include a small sample size and lack of clinical validation. In this study, we utilized bioinformatics methods to investigate and predict miRNA target genes and associated biological signalling pathways relevant to type 2 CRS. This study represents our preliminary experiment. However, we intend to conduct clinical validation through cohort studies focusing on the screened differential miRNAs.

## Conclusions

Endogenous miRNAs were differentially expressed among healthy individuals, patients with CHF and patients with CRS. Prediction of differential miRNA target genes and through GO function and KEGG pathway analysis, the molecular mechanisms of CRS may be revealed. Circulating miRNAs may contribute to the diagnosis of CRS, and further and larger studies are needed to enhance the robustness of our findings.

## Data Availability

The datasets used and/or analysed during the current study available from the corresponding author on reasonable request.
